# Touch Satiety: Differential Effects of Stroking Velocity on Liking and Wanting Touch Over Repetitions

**DOI:** 10.1371/journal.pone.0113425

**Published:** 2014-11-18

**Authors:** Chantal Triscoli, Rochelle Ackerley, Uta Sailer

**Affiliations:** 1 Dept. of Psychology, University of Gothenburg, Gothenburg, Sweden; 2 Clinical Neurophysiology, Sahlgrenska University Hospital, Gothenburg, Sweden; 3 Dept. of Physiology, Institute for Neuroscience & Physiology, University of Gothenburg, Gothenburg, Sweden; Knox College, United States of America

## Abstract

A slow, gentle caress of the skin is a salient hedonic stimulus. Low threshold, unmyelinated C-tactile afferents fire preferentially to this type of touch, where slow (<1 cm/s) and fast (>10 cm/s) stroking velocities produce lower firing frequencies and are rated as less pleasant. The current aim was to investigate how the experience of tactile pleasantness changes with repeated exposure (satiety to touch). A further aim was to determine whether tactile satiety varied with different stroking velocities. The experimental paradigm used a controlled brush stroke to the forearm that was delivered repeatedly for ∼50 minutes. In Experiment 1, brush strokes were administered at three different velocities (0.3 cm/s, 3 cm/s and 30 cm/s), which were presented in a pseudo-randomised order. In Experiment 2, brush strokes were applied using only one velocity (either 3 or 30 cm/s). After each stroke, the participants rated both subjective pleasantness (liking) and wanting (the wish to be further exposed to the same stimulus) for each tactile sensation. In Experiment 1, both pleasantness and wanting showed a small, but significant, decrease over repetitions during stroking at 3 cm/s only, where the mean values for pleasantness and wanting were similar. Conversely, slower (0.3 cm/s) and faster (30 cm/s) stroking showed no decrease in ratings over time, however pleasantness was rated higher than wanting. In Experiment 2, both pleasantness and wanting showed a significant decrease over repetitions for both applied velocities, with a larger decrease in ratings for stroking at 3 cm/s. In conclusion, satiety to touch occurred with a slow onset and progression, where pleasantness and wanting ratings to stroking at 3 cm/s were affected more than at the slower or faster velocities. Tactile satiety appears to differ compared to appetitive and olfactory satiety, because the hedonic and rewarding aspects of touch persist for some time.

## Introduction

Satiety, the decrease in reward value due to repeated exposure, has been studied to the greatest extent in the olfactory and gustatory literature. Findings regarding satiety from eating food have shown that the subjective pleasure derived from of an ingested food and the motivation to obtain more of the same food, decline with consumption [1]–[5]. Studies have found that the perceived pleasantness of food decreases even faster when repeatedly exposed to the same food, a phenomenon called sensory-specific satiety [6]–[9]. In olfaction, it has been speculated that the pleasantness of an appealing smell can decrease after repeated exposure [10] and it can even shift towards aversion [11]. However, it has also been found that using a constant olfactory stimulus frequency and strength, the hedonic value of pleasant odours is maintained over repeated exposures [12].

For visual stimuli, it has been shown that the repeated presentation of erotic visual material leads to a decrease in physiological arousal [13]. This can be considered a case of habituation, which is characterised by a decline in the responsiveness to a stimulus [14]. Satiety is a different process to habituation, as satiety occurs when a stimulus is no longer perceived as pleasant after repeated exposure, therefore is not desired anymore and/or becomes aversive [9]. To our knowledge, no previous investigations have looked at how repeated exposure to affective touch is experienced, and more specifically, whether and how the pleasantness of touch satiates. Affective touch is signalled, in part, by unmyelinated, slowly-conducting (∼1 m/s) low-threshold mechanoreceptors, called C-tactile (CT) afferents, which are only found in the hairy skin of the body [15]–[20]. Affective, emotional touch can be distinguished from discriminative, conscious touch by the speed of the conduction of the afferent information. Discriminative touch requires the activation of myelinated, fast-conducting, Aβ low-threshold mechanoreceptors for the perception of tactile qualities like force, velocity, texture and pressure [21]. Human microneurography experiments have found that CT afferents respond well to slow, gentle stroking, with their highest firing frequencies occurring to strokes within the velocity range 1–10 cm/s [15], [22]. The stroking velocity response curve gained in CT firing frequency, where slower and faster velocities produce lower firing frequencies, is mirrored in psychophysical judgments of the pleasantness of the same stroking stimulus [15], [22]. In combination with imaging studies showing similar effects of stroking velocity on insular responses [23], the CT system appears to code the positive aspects of affective touch.

CT afferents have been found to fatigue over time, where their responses become depressed after repeated exposure to tactile stimuli. This high dependency to previous touch has been explored in animals (in C-low threshold mechanoreceptors, CLTMs) [24]–[26] and to some extent in humans [16], [20]. In humans, repeated, supra-threshold tactile stimuli can render CTs less responsive, although they do continue to fire to touch [16], [20]. In animals, the depression of CLTM responses appears to be much longer-lasting [26], which may indicate between-species differences. The myelinated mechanoreceptors show substantially little, if any, fatigue to repeated tactile stimulation [27]. Thus, it is of interest to study the effects of possible fatigue in CT afferents and whether it occurs more readily at a stroking velocity that optimally activates CT afferents as compared to using sub-optimal activation.

The subjective experience of a pleasant sensory stimulus relates to the concept of reward, which is characterised by the two inter-related components of “liking” and “wanting” [28]. “Liking” is an affective reaction to the hedonic evaluation of a reward, which is inherently connected to the derived pleasure or pleasantness [29]–[35]. On the other hand, the concept of “wanting” corresponds to the motivational value and incentive attributed to the reward [36]–[38]. There has been an attempt to dissociate liking and wanting in humans, where pharmacological manipulations in the so-called “hotspots” of the limbic structures (particularly nucleus accumbens and ventral pallidum) have been shown to alter wanting without affecting liking, and vice versa [28], [36], [39]. Moreover, liking, wanting and intensity have been shown to be independent of sensory modality in a study in chocolate cravers and non-cravers using functional magnetic resonance imaging [40]. The primary sensory cortical areas (visual, gustatory) showed no differences between cravers; however, the orbitofrontal cortex, ventral striatum and pregenual cingulate cortex showed specific stimulus activations, which were further linked to the degree of craving (wanting). Additionally, the amount of pregenual cingulate cortex activity was correlated with the rated pleasantness (liking) and intensity of the stimulus, but not with wanting [40]. Another attempt to assess liking and wanting through separate measurement techniques showed a partial dissociation between these concepts for generic food categories [41]. However, dissociating the concepts of liking and wanting remains a challenge, as it is likely that there are separate neural correlates in specific situations for liking and wanting, but the concepts are inextricably inter-related and depend on personal preferences [42].

The aim of the present work was to determine whether and how the evaluation of repeatedly-presented, pleasant tactile stimuli (brush strokes) changed with regard to the pleasantness sensation (“liking”) and the wish to be exposed further to them (“wanting”), and whether this differed with the velocity of the moving stimulus. Based on the phenomenon of sensory-specific satiety (in taste), we hypothesised that the rate of change in liking and wanting would be lower for varying-velocity brush strokes (Experiment 1, within-subjects) than for non-varying stimuli (constant velocity brush strokes; Experiment 2, between-subjects). Moreover, since CTs have been shown to fatigue, we predicted that the pleasantness of stroking at a CT-optimised stroking velocity (3 cm/s), should decrease more rapidly over repetitions than at the other velocities. Moreover, considering the strong connection between the reward system and aspects of liking and wanting, questionnaires were administered to evaluate personality traits such as reward sensitivity, approach motivation towards rewarding stimuli and inclination to tactile experiences. We predicted that those individuals who scored highly on these traits would show a lesser decrease in pleasantness ratings over stroking repetitions.

## Materials and Methods

### Experiment 1 (Within subjects, different stroking velocities)

Experiment 1 was designed to test how the variation of brush stroke velocity, delivered over repetitions, leads to a change in the pleasantness (liking) and wanting of the stroke.

#### Participants

In total, 12 participants (6 males), aged between 19 and 28 years, took part in the study. The study was approved by the ethics committee of the University of Gothenburg and the participants received financial compensation for participation in the study (200 SEK per hour, ∼22 €). Written informed consent was obtained from all subjects prior to their participation.

#### Experimental setting and procedure

After receiving a written explanation of the experiment which included a description of the experimental setting and the instructions on how to make the ratings (see below), and after having giving written informed consent, the participants sat in a chair in front of a computer screen with their left arm immobilised comfortably in a vacuum cast. The participants wore headphones to minimise noise, and occluding glasses that blocked their peripheral vision, shielding them from seeing the tactile stimulation. Each participant was stroked on their left dorsal forearm via a custom-built robotic device, which delivered highly replicable force and velocity stimuli. The strokes were performed using a 50 mm wide flat, soft water-colour brush made of fine, smooth, goat's hair. The brush was fixed to a rotary tactile stimulator (RTS; Dancer Design; St Helen's, UK) robot driven by LabVIEW software (National Instruments; Austin, TX). Brush strokes on the participant's forearm were given at a calibrated normal force of 0.4 N, which is sufficient to activate low-threshold mechanoreceptors. One trial consisted of 5 back-and-forth brush strokes, where the brush traversed a distance on the skin of ∼10 cm for each stroke. After each trial, participants rated the sensation on two subsequently presented visual analogue scales (VAS) using a touch-screen tablet computer (iPad, Apple; Cupertino, CA), which was fixed to a table in front of the participant.

In the first VAS, the participants were asked to answer the question: “How pleasant was the brushing?”. This scale had the endpoints “not at all pleasant” (with an output of −10) on the left and “very pleasant” (with an output of +10) on the right, and was intended to measure the concept of “liking”. In the second VAS, which occurred directly after the first one, the participants were asked to answer the question: “How much do you want another stroke of the same velocity?”. This scale had the endpoints “not at all” (with an output of −10) on the left and “very much” (with an output of +10) on the right, and was intended to measure the concept of “wanting”. The participant had 5 s to complete both VAS; each VAS disappeared as soon as the participant had given the rating, or timed-out if the answers were not given within the allotted time. The output that corresponded to the middle of both these scales (i.e. zero) was used as a middle point to distinguish between pleasant and unpleasant in the first scale and between low and high wanting in the second scale.

Three different brush-stroke velocities were used: “slow” (0.3 cm/s), “medium” (3 cm/s) and “fast” (30 cm/s), with 40 repetitions of each. The three velocities were chosen as they activate CT fibres differently, and the firing rate of CTs correlates with tactile pleasantness [15], [22]. The peak firing frequency of CT fibres in the hairy skin (forearm) occurs at stroking of 1–10 cm/s, which corresponds to the “medium” velocity in the present study. Stroking at the “slow” velocity produces many impulses from CT afferents, but at a lower frequency; conversely, stroking at the “fast” velocity gives only a few impulses, but also at a lower firing frequency [15], [22]. In the present experiment, the three velocities were delivered in a pseudo-randomised order across the participants, so that the same velocity would occur maximally twice in a row. In summary, there were 40 trials for each of the three velocities, adding up to 120 trials in total, which lasted for ∼50 minutes.

After the experiment, the participants filled in three different questionnaires, the Behavioural Inhibition and Activation Systems Scale (BIS/BAS; [43]), the Temporal Experience of Pleasure Scale (TEPS; [44]) and the Need for Touch Scale [45]. The BIS/BAS scale consists of 24 items that measure behavioural approach or positive affect in response to reward (BAS), and avoidance or negative affect in response to punishment (BIS) [46]. The BAS-scale is divided into several subscales: BAS Drive, measuring the degree to which reward outcomes guide subsequent behaviours; BAS Fun Seeking, the degree to which a person is oriented towards novelty seeking; and BAS Reward Responsiveness, measuring the degree to which a person derives pleasure from reward [47]. The TEPS is a measure of individual trait dispositions in both Anticipatory (reward responsiveness and imagery) and Consummatory (openness to different experiences and appreciation of positive stimuli) experiences of pleasure. The Need for Touch scale is designed to measure individual differences in preference for touch and the degree to which a person has a “need” for tactile input. The scale is divided into two sub-scales, the Autotelic dimension, which involves a hedonic-oriented response towards sensory stimulation itself, and the Instrumental dimension, which refers to aspects of pre-purchase touch that reflect outcomes directed to a purchase goal of any kind of commercially-available products. Participants were instructed that there were no right or wrong responses, only subjective ones related to each one's personal experiences. All participants completed all the questionnaires, the results of which are presented in Experiment 2.

#### Statistical analysis

Statistical analyses were made using SPSS Statistics version 21 (IBM; Chicago, USA). To determine whether the ratings changed significantly over the repetitions, linear regression analyses were performed separately with “pleasantness” and “wanting” as the outcome variables and the number of repetitions as the predictor, at each stroking velocity. To determine general differences between pleasantness and wanting, the VAS ratings for each participant were averaged, obtaining mean values for “pleasantness” and “wanting” at each velocity. The mean pleasantness and wanting ratings were submitted as dependent variables in a multivariate analysis of variance (MANOVA), with velocity (0.3 cm/s, 3 cm/s and 30 cm/s) as the fixed factor. Greenhouse-Geisser corrections were used to adjust for violations of sphericity. The level of significance was set to p<0.05. Post-hoc t-tests, corrected for multiple comparisons using Tukey's method, were used to explore interaction effects. Separate paired-sample t-tests were conducted at each velocity to compare the mean level of pleasantness and wanting ratings. Finally, to investigate the relationship between pleasantness and wanting, a further linear regression analysis was performed for all the velocities together with “wanting” as the outcome variable and “pleasantness” as the predictor, in order to determine whether the pleasantness ratings could significantly predict the subsequent wanting ratings.

### Experiment 2 (Between subjects, same stroking velocity)

To determine whether the liking and wanting of touch decreased when the stimulus is not varied, we conducted a second experiment. Here, the “medium” stroking velocity of 3 cm/s was applied to one group of participants, and the “fast” 30 cm/s stroking was applied to a different group. The 0.3 cm/s velocity was not used because the ratings obtained for the velocities of 0.3 and 30 cm/s produced very similar outcomes in Experiment 1. Experiment 2 was designed to test how two different brush stroking velocities to two different groups of subjects led to a change in the pleasantness and wanting of touch, when delivered repeatedly.

#### Participants

In total, 17 individuals (6 males), aged between 19 and 66 years, participated in the experiment. One participant differed substantially from the others in terms of age (66 years); however, we did not exclude her from the study because she was deemed healthy and calculating all the analyses without this participant did not change the results. Thus, her behaviour was similar to that of the other participants. None of the participants had taken part in Experiment 1. The study was approved by the ethics committee of the University of Gothenburg and the participants received financial compensation for participation in the study (200 SEK per hour, ∼22 €).

#### Experimental setting and procedure

The experimental setting and procedure of Experiment 2 were identical to those of Experiment 1 in terms of the left forearm stimulation; however, each participant was repeatedly stroked with one velocity only. One group of participants (N = 9) were stroked at 30 cm/s for a duration of 50 minutes (to keep the same stroking duration as in Experiment 1), resulting in 267 trials. One participant terminated the experiment after 169 trials because she found the ongoing stimulation (at 30 cm/s) intolerable. However, the rating data of this participant was included in the analyses. Another group (N = 8) was stroked at 3 cm/s for a duration of 50 minutes, resulting in 120 trials. After the experiment, the participants filled in the same questionnaires used in Experiment 1 (BIS/BAS, TEPS and “Need for touch” scales). All participants completed all the questionnaires.

#### Statistical analysis

To determine whether the ratings changed significantly across repetitions, linear regression analyses were performed separately with “pleasantness” and “wanting” as the outcome variables and the number of repetitions per velocity as the predictor. A paired-sample t-test was conducted to compare the mean level of pleasantness and wanting at the 3 cm/s stroking velocity, and at the 30 cm/s velocity. The VAS-ratings for each participant were averaged, obtaining mean values of “pleasantness” and “wanting” for both velocities, i.e. for both groups. Furthermore, a MANOVA with the factor “stroking velocity” (3 cm/s and 30 cm/s) and the mean ratings of pleasantness and wanting as dependent variables was used to determine whether the two reward aspects were evaluated differently depending on velocity.

As for Experiment 1, in order to investigate the relationship between pleasantness and wanting, a further linear regression analysis was performed for all the velocities together with “wanting” as the outcome variable and “pleasantness” as the predictor, in order to determine whether the pleasantness ratings could significantly predict the subsequent wanting ratings.

### Comparisons between Experiments 1 and 2

It was of interest to compare the end ratings for both pleasantness and wanting between Experiments 1 and 2, for stroking at 3 and 30 cm/s. Independent t-tests were used to see whether the constant-velocity stroking for 50 minutes (Experiment 2) produced significantly different pleasantness and wanting ratings, respectively, compared to the mixed-velocity stroking (Experiment 1).

### Correlations between the Ratings and Touch Questionnaires

The mean pleasantness ratings were correlated with the mean wanting ratings, the regression slopes and the scores of the questionnaire scales, using Pearson's correlation. For this analysis, the participants from Experiments 1 and 2 were pooled together (N = 29). Analogous correlations were calculated for the mean wanting ratings.

## Results

### Experiment 1 (Within subjects, different stroking velocities)

**Figure 1:**
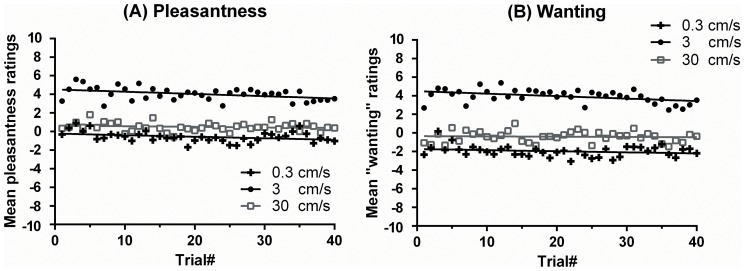
Ratings over time for (A) pleasantness and (B) wanting for stroking at different velocities in Experiment 1 (within-subjects). Significant decreases were found for the pleasantness and wanting of stroking at 3 cm/s over stroking repetitions; this decrease over time was not found for stroking at 0.3 and 30 cm/s. Furthermore, the pleasantness and wanting of touch was significantly higher for stroking at 3 cm/s, as compared to 0.3 or 30 cm/s.

The slope of the ratings was analysed across trials, to determine the change in pleasantness and wanting over time. A small, but significant, decrease in pleasantness ratings was found for stroking at 3 cm/s, but not for stroking at 0.3 or 30 cm/s (3 cm/s: *t*(477) = −2.04, standard error (SE) = 0.01, R = 0.09, Beta = −0.09, *p* = .042; 0.3 cm/s: *t*(477) = −1.06, SE = 0.01, R = 0.05, Beta = −0.05, *p* = .290; 30 cm/s: *t*(478) = −0.50, SE = 0.01, R = 0.02, Beta = −0.02, *p* = .617), as shown in Figure 1A. The ratings decreased on average by 0.02 VAS-points after each 3 cm/s trial. Analogous results were found for wanting touch. The mean wanting ratings decreased slightly but significantly with the number of repetitions, only for the stroking velocity of 3 cm/s but not for 0.3 or 30 cm/s (3 cm/s: *t*(476) = −2.05, SE = 0.01 R = 0.09, Beta = −0.09, *p* = .041; 0.3 cm/s: *t*(474) = −0.62, SE = 0.01, R = 0.03, Beta = −0.03, *p* = .537; 30 cm/s: *t*(474) = 0.22, SE = 0.01, R = 0.01, Beta = −0.01, *p* = .823), as shown in Figure 1B.

**Figure 2:**
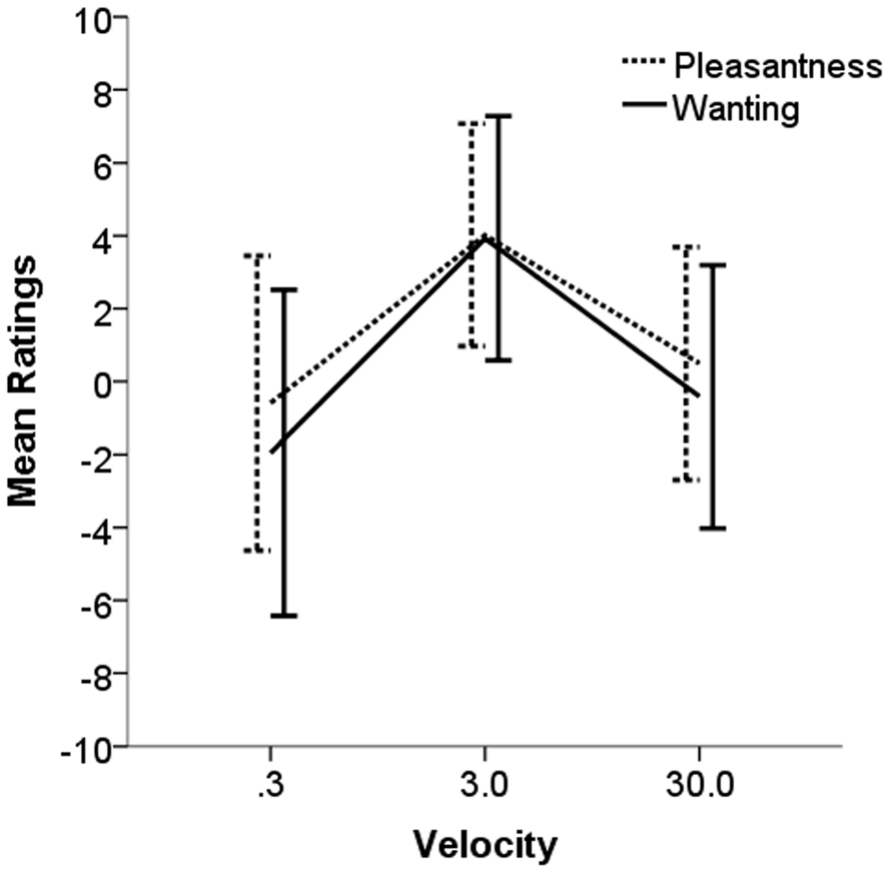
Mean pleasantness and wanting ratings for the three different stroking velocities in Experiment 1 (within subjects). Stroking at 3 cm/s was rated as significantly more pleasant and wanted than stroking at 0.3 or 30 cm/s. However, there was no significant difference between the value of pleasantness or wanting of touch at 3 cm/s stroking, whereas pleasantness was significantly higher for stroking at 0.3 and 30 cm/s.

Overall, pleasantness ratings for stroking at 3 cm/s were significantly higher than ratings for stroking at 0.3 and 30 cm/s (3 cm/s: *M* = 4.04, *SD* = 3.04; 0.3 cm/s: *M* = −0.56, *SD* = 4.02; 30 cm/s: *M* = 0.50, *SD* = 3.20) (Figure 2). The same tendencies were found for wanting; ratings for stroking at 3 cm/s were significantly higher than ratings from stroking at 0.3 and 30 cm/s (3 cm/s: *M* = 3.96, *SD* = 3.34; 0.3 cm/s: *M* = −1.98, *SD* = 4.50; 30 cm/s: *M* = −0.42, *SD* = 3.62) (Figure 2).

The velocity of stroking had a significant effect on the pleasantness and wanting of touch (MANOVA Pillai's Trace statistic; V = 0.45, *F*(4, 66) = 12.06, *p*<.001). The subsequent univariate ANOVAs on the ratings showed that there was a significant effect of stroking velocity on both pleasantness (*F*(2, 17.33) = 8.93, *p* = .001) and wanting (*F*(2, 28.23) = 12.62, *p*<.001) of touch. Tukey's corrected pairwise comparisons revealed several significant differences between velocities in the pleasantness and wanting ratings: the pleasantness ratings were significantly higher for stroking at 3 cm/s than for both 0.3 cm/s (mean difference = −4.58, SE = 1.14, *p* = .001) and 30 cm/s (mean difference = 3.54, SE = 1.14, *p* = .010); conversely, the pleasantness ratings for stroking at 0.3 cm/s and 30 cm/s were not significantly different (mean difference = 1.04, SE = 1.14, *p* = .632). The same tendencies were found for wanting: the ratings were significantly higher for stroking at 3 cm/s than for stroking at 0.3 cm/s (mean difference = 5.92, SE = 1.22, *p*<.001) and for 30 cm/s (mean difference = 4.38, SE = 1.22, *p* = .003); conversely, the wanting ratings for stroking at 0.3 cm/s and 30 cm/s were not significantly different (mean difference = 1.54, SE = 1.22, *p* = .431) (Figure 2).

The mean levels of the ratings were compared within each stroking velocity, to assess whether there were differences between the levels of pleasantness and wanting. Significantly higher values were found for pleasantness, rather than wanting, for stroking at 0.3 cm/s (*t*(11) = 2.94, *p* = .019) and 30 cm/s (*t*(11) = 2.50, *p* = .030) (Figure 2). On the other hand, the pleasantness and wanting ratings were not significantly different for stroking at 3 cm/s, (*t*(11) = 0.29, *p* = .777).

The linear regression with “pleasantness” as the predictor and “wanting” as the outcome variable, showed that wanting rating could be predicted directly from the pleasantness rating, with a significant positive trend (*t*(1426) = 79.24, SE = 0.01, R = 0.90, Beta = 0.90, *p*<.001). Wanting explained the majority of the variance in pleasantness (R^2^ = 0.82), showing that pleasantness and wanting were intricately related.

### Experiment 2 (Between subjects, same stroking velocity)

**Figure 3:**
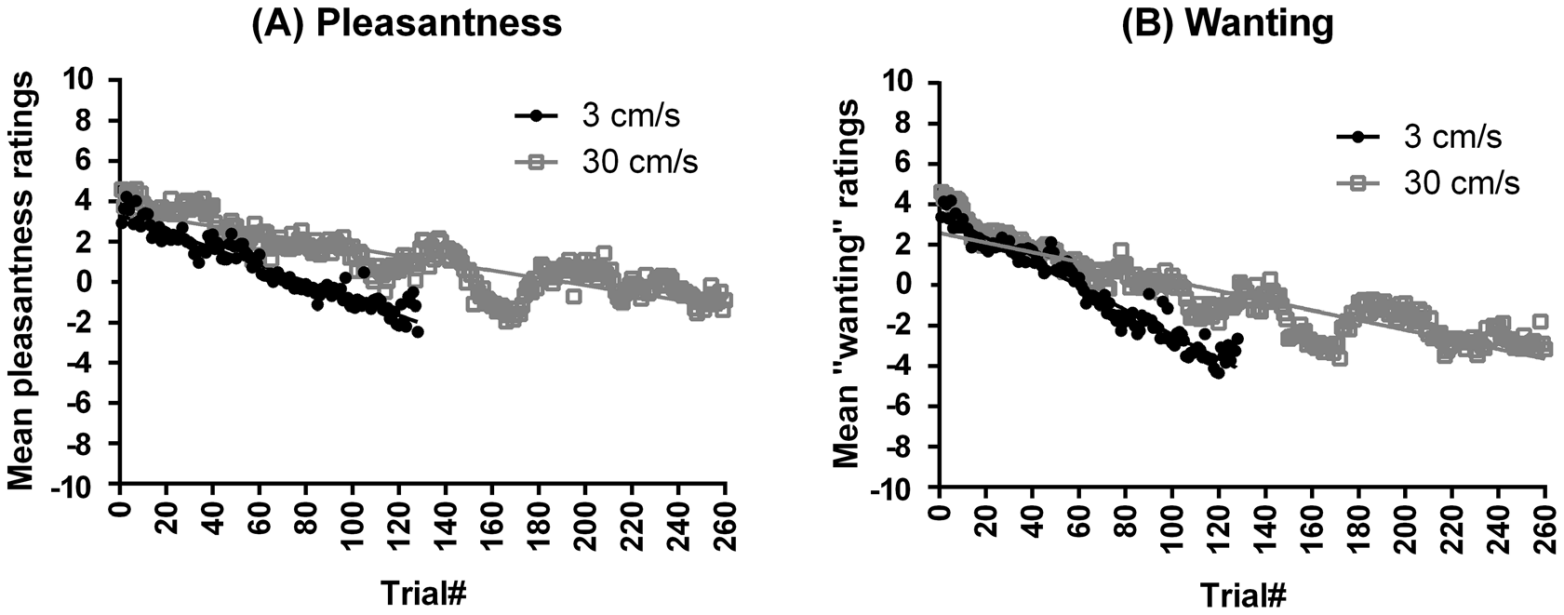
Decrease over repetitions in the pleasantness and wanting ratings for the two different stroking velocities in Experiment 2 (between-subjects). Significant decreases were found over time in pleasantness and wanting ratings for stroking at 3 cm/s and at 30 cm/s in the separate groups of participants. Pleasantness and wanting decreased at a faster rate for stroking at 3 cm/s, as compared to at 30 cm/s.

A significant decrease in pleasantness ratings with the number of repetitions was found for both stroking velocities (3 cm/s: *t*(934) = −13.04, SE = 0.00, R = 0.40, Beta = −0.40, p<.001; 30 cm/s: *t*(2313) = −21.54, SE = 0.00, R = 0.41, Beta = −0.41, *p*<.001) (Figure 3A). Analogous results were found for wanting, where the mean wanting ratings decreased significantly with the number of repetitions for both stroking velocities (3 cm/s: *t*(921) = −17.62, SE = 0.00, R = 0.51, Beta = −0.51, p<.001; 30 cm/s: *t*(2295) = −23.41, SE = 0.00, R = 0.44, Beta = −0.44, *p*<.001) (Figure 3B).

Overall, the mean pleasantness ratings were in the slightly negative range for both velocities (3 cm/s: *M* = −0.74, *SD* = 3.00; 30 cm/s: *M* = −1.12, *SD* = 2.44) (Figure 3A). Similarly, the mean wanting ratings were in the slightly negative range for both velocities (3 cm/s: *M* = −0.22, *SD* = 3.12; 30 cm/s: *M* = −0.56, *SD* = 2.96) (Figure 3B). The paired samples t-tests comparing the mean levels of pleasantness and wanting per stroking velocity revealed that the ratings for pleasantness were higher than the ratings for wanting, both when stroking was applied at 3 cm/s (*t*(7) = 2.72, *p* = .030) and 30 cm/s (*t*(8) = 5.11, *p* = .001).

The comparison of pleasantness and wanting across stroking velocities showed no significant main effect of velocity on the pleasantness and wanting of touch (MANOVA Pillai's Trace statistic; V = 0.70, *F*(2, 14) = 1.21, *p* = .327). Thus, the pleasantness ratings were similar for 3 cm/s and 30 cm/s stroking (mean difference = −0.20, SE = 0.66), as were the wanting ratings (mean difference = 0.17, SE = 0.74).To further investigate the relationship between pleasantness and wanting as related concepts, a linear regression with “pleasantness” as the predictor and “wanting” as the outcome variable gave a similar result to Experiment 1, showing that wanting could be predicted from pleasantness, in a significant positive trend (*t*(3215) = 106.03, SE = 0.01, R = 0.88, Beta = 0.88, *p*<.001). Again, wanting explained the majority of the variance in pleasantness (R^2^ = 0.78).

### Comparisons between Experiments 1 and 2

Independent t-tests were used to explore whether differences in pleasantness and wanting ratings existed at the end of constant-velocity stroking in Experiment 2, compared to the mixed-velocity stroking in Experiment1 (note that both experiments were of ∼50 minutes duration). Pleasantness end-ratings for the velocity of 3 cm/s were significantly higher in Experiment 1 where only 40 trials at 3 cm/s were presented, than in Experiment 2 where 120 trials were presented (mean difference = 5.66, SE = 1.60; *t*(16) = 3.55, *p* = .003). The same was true for the end ratings of wanting (mean difference = 7.86, SE = 1.74; *t*(16) = 4.50, *p*<.001). There was no significant difference between the end ratings for pleasantness (mean difference = 1.46, SE = 1.52; *t*(18) = 0.96, *p* = .349) or wanting (mean difference = 2.64, SE = 1.52; *t*(18) = 1.73, *p* = .100) for stroking at 30 cm/s between Experiment 1 (total 40 trials at 30 cm/s) and Experiment 2 (267 trials).

### Correlations between the Ratings and Touch Questionnaires

Significant negative correlations were found between the BAS Fun Seeking and the mean pleasantness ratings (*r*(27) = −0.40, *p* = .034,), as well as between BAS Fun Seeking and the mean wanting ratings (*r*(27) = −0.42, *p* = .022). In addition, a significant positive correlation was found between all the mean pleasantness and mean wanting ratings (*r*(27) = 0.91, *p*<.001). No significant correlations were found between the slopes of pleasantness and wanting touch and the questionnaire scales. Correlations among different instruments are not reported.

## Discussion

To our knowledge, this is the first study investigating whether the experience of tactile stimuli “satiates”. Overall, small, but significant, decreases in both pleasantness and wanting were found consistently with stroking at 3 cm/s over time. However in Experiment 1 (within-subjects, different stroking velocities), only the ratings to 3 cm/s stroking showed any satiety, whereas in Experiment 2 (between-subjects, same stroking velocity) some satiety occurred for both stroking velocities. Nevertheless, even after 50 minutes of stroking in both experiments, the sensation was never rated as really unpleasant. This implies that pleasant touch is rather robust against satiety, unlike gustatory stimuli. In addition, we attempted to distinguish between two aspects of reward: the perception of pleasantness (liking) and the wish to be touched with the same stimulus again (wanting). The results from both experiments demonstrate that, as for the other widely studied sensory modalities of taste and olfaction [2], the pleasantness and wanting of touch were inherently related, as in all analyses, pleasantness could be predicted from wanting and vice versa.

### Experiment 1 (Within subjects, different stroking velocities)

Pleasantness (liking) and wanting ratings only decreased for stroking at 3 cm/s, which is within the optimal range (1–10 cm/s) for CT fibre activation [15], [22]. Although statistically significant, the decrease was small. In contrast to stroking at the CT-optimal velocity, ratings for the slower and faster velocities did not show any significant decrease over repetitions. None of the velocities applied were rated as very unpleasant, even after 40 repetitions. Taken together, this may suggest that satiety in the domain of touch may take much longer to occur than for other primary rewards such as food [6], [8], [9], [48], [49], or may not occur at all.

In accordance with previous studies [15], [22], [38], [50], [51], pleasantness ratings were higher for the CT-optimal stroking velocity of 3 cm/s than for the slower velocity of 0.3 cm/s and the faster velocity of 30 cm/s. The differences found in the pleasantness of stroking over different velocities are thought to be underpinned by the CT system. The firing frequency of CT afferents is highest at stroking velocities of 1–10 cm/s, and the pleasantness ratings of the same stimuli mirror the CT response; whereas the myelinated afferents, which contribute more to discriminative touch, show no correlation with pleasantness ratings [15], [22]. The decrease in pleasantness and wanting for repetitive stroking at 3 cm/s velocity may be linked to fatigue in CT afferents, where repetitive touch can decrease the activity of CTs to subsequent touch [16], [20]. Fatigue in CT afferents would produce reduced firing, which could have led to a selective modulation in the appraisal of pleasant touch over repetitions. The mechanism for CT fatigue at the peripheral receptor and whether CTs fatigue more to their optimal stimulus are unknown; however, we cannot disregard that a decline in the pleasantness and wanting of touch from the repetitive stroking at 3 cm/s may also reflect central changes in touch perception. It seems that the degree of activity in myelinated low-threshold mechanoreceptive afferents over stroking repetitions would have negligible effects on touch satiety because these afferents, in general, show no or minimal fatigue to repeated stimulation [27]. Therefore, the tactile input from the myelinated mechanoreceptive afferents over repetitions is largely unaffected, which is consistent with the lack of touch satiety from stroking at 0.3 or 30 cm/s.

The mean pleasantness and wanting ratings varied in level with the stroking velocity. This indicated that pleasantness and wanting could be distinguished as different concepts; however, pleasantness could be significantly predicted from wanting, showing that the two measures were highly related. This may be due to the fact that the order of VAS ratings was not varied and that they directly followed each other. Thus, the relationship between pleasantness and wanting needs to be interpreted carefully, in particular since further validation of the two measures is required to establish their contribution in touch satiety.

It is possible that the small decrease over repetitions for the pleasantness and wanting of touch at the CT-optimal velocity, and the constancy in the ratings for the other two velocities occurred because the participants randomly experienced all three velocities in the same experiment. This can be compared to the domain of eating, where variation in food intake delays the development of satiety [52], as compared to when the same food is ingested, as in sensory-specific satiety [6]–[9]. To determine whether the pleasantness and wanting of touch decreases when the stimulus was not varied, we conducted Experiment 2.

### Experiment 2 (Between subjects, same stroking velocity)

Experiment 2 investigated whether the pleasantness of tactile stimuli (liking) and the wish to be touched again (wanting) changed with repeated exposure, but this time using the same tactile stimulus over all the repetitions (i.e. one single stroking velocity for each group of participants to induce sensory-specific satiety). Both pleasantness and wanting decreased significantly and strongly for the velocity of 3 cm/s, and also for the stroking velocity of 30 cm/s. Thus, for both groups, the perceived pleasantness of a tactile stimulus decreased with repetitions when the stroking velocity was kept constant. However, the 30 cm/s stroking condition was still perceived as neutral after 50 minutes of stimulation, unlike the 3 cm/s condition, which was rated as slightly less pleasant.

The more rapid decrease in tactile pleasantness and wanting during single-velocity stroking demonstrates possible sensory-specific satiety in touch. The taste literature has shown that satiety can occur to food; however, with no gustatory variation, satiety occurs faster [6]–[9], [52], although it may depend on the specific food itself [53]. In the domain of taste, satiety has been defined as the physiological state that occurs during food consumption and terminates the food intake [1]. Thus, it seems that satiety occurs when the stimulus is experienced as aversive (i.e. physically cannot eat more) and has the function to stop the stimulation. We may conclude that satiety occurred for the velocity of 3 cm/s, when the ratings became slightly unpleasant, but probably less so for the faster velocity, given that the stimulation was still evaluated in the positive, pleasant range. As further support, wanting (i.e. the wish to be touched again) decreased, irrespective of the velocity of the stroke. Nevertheless, the decrease for 3 cm/s ended up in the negative range faster than the decrease for 30 cm/s.

In Experiment 2, both velocities showed similar mean values for overall pleasantness and wanting. Thus, in applying the same tactile stimulus over many repetitions, there were no differences between the two groups. This finding contradicts the literature on the CT-optimal velocity range [15], [22], [38], [50], [51], where 3 cm/s is typically perceived as the most pleasant velocity. The reason for this difference is presumably that each group was submitted to only one velocity, which made it impossible to compare the different stroking velocities. In the same way, stimulus intensity has been found to affect a conditioned response when it varied within-subjects, but not between-subjects [54]. It therefore seems that the modulation and subsequent evaluation of perceived pleasantness is at least partially influenced by the memory of the previous ratings which provides an anchor relative to which the subsequent ratings were compared [50], [51].

### Considerations

The present work describes the phenomenon of touch satiety. The question arises as to whether touch satiety is a peripheral and/or central mechanism. In Experiments 1 and 2, the participants were stroked for ∼50 minutes, which may have led to satiety through cognitive factors such as boredom. From the current results, it appears that both peripheral and cognitive mechanisms are likely to play a part in the degree of touch satiety. The appraisals of very slow and fast touch were different from the appraisals of the CT-optimal stroking at 3 cm/s, which indicates that CT-innervated skin may be more sensitive to tactile satiety, pointing towards a peripheral mechanism. Conversely, Experiment 2 showed that sensory-specific (single-velocity) stroking gave increased satiety, which is more likely to be a central effect.

The present study used a robotic device to apply the controlled stroking stimuli. The use of a robotic, rather than a more ecologically valid “stimulator” such as a human hand, may reduce the extent to which we can generalise our results; however, the literature demonstrates that a robotic brush stroke is experienced as extremely pleasant [15], [22], [38], [50], [51], [55]. Furthermore, a recent study showed that the pleasantness derived from a brush stroke delivered by hand or by a robot was comparable [38]. In the present study, a robotic stimulator was used to ensure that a precise, controlled and repeatable stroke was delivered each time, especially as different stroking velocities were used. Although more ecologically valid, the use of human touch could have influenced pleasantness in an unpredictable way (i.e., the hands could have changed in temperature and moisture over time, or the participant could have liked or disliked the experimenter). Overall, we found that touch satiety did occur to some extent with the robotic stimulator, after some time. If human touch is even more pleasant than robotically-delivered touch, satiety may take even longer to occur. However, we predict that central mechanisms (e.g. the relationship between the touched and the person doing the touching) would play a critical role during inter-personal touch and its satiation.

Both experiments showed that pleasant touch leads to tactile satiety after repeated exposure, but this took some time. During social interactions, the repeated exposure to gentle, stroking touch such as a human caress may lose its beneficial value over repetitions, and thus affect the perceived pleasantness and the willingness to be touched again. This may be related to the relevance of touch, where briefer social interactions may convey more meaningful information. The present line of inquiry could be further extended by investigating whether touch satiety differs between cultural populations (e.g. those with high inter-personal contact) or certain clinical populations (e.g. autism), as well as investigating the effect of hormones (e.g. oxytocin). Future experiments using microneurography aim to record directly from tactile afferents in the hairy skin of the arm to assess whether the extent to which peripheral receptor fatigue is affected with this type of repetitive touch.

## Conclusions

Our results show that both the pleasantness (liking) and wanting of touch decrease with repeated stimulation, but that this effect was subject to differences in the stroking paradigm. It was clear that for the CT-optimal velocity of 3 cm/s, a small but significant effect of touch satiety occurred. Since this stroking velocity is the most similar to a human caress, one may speculate that its hedonic value may decrease when it is given repeatedly in exactly the same way. Moreover, our results suggest for the first time that the phenomenon of sensory-specific satiety, widely studied for the sense of taste, may also occur in the domain of touch. This was particularly found during the like-caress stroking velocity of 3 cm/s; however, tactile satiety only seems to appear when the stimulation is ongoing for quite a long time.

## References

[1] Bellisle F, Drewnowski A, Anderson GH, Westerterp-Plantenga M, Martin CK (2012) Sweetness, satiation, and satiety. J Nutr 142: 1149S–1154S. 22573779 10.3945/jn.111.149583

[2] Havermans RC, Janssen T, Giesen JC, Roefs A, Jansen A (2009) Food liking, food wanting, and sensory-specific satiety. Appetite 52: 222–225. 18951934 10.1016/j.appet.2008.09.020

[3] Nasser J (2001) Taste, food intake and obesity. Obes Rev 2: 213–218. 12119992 10.1046/j.1467-789x.2001.00039.x

[4] Rolls ET (2005) Taste, olfactory, and food texture processing in the brain, and the control of food intake. Physiol Behav 85: 45–56. 15924905 10.1016/j.physbeh.2005.04.012

[5] Sorensen LB, Moller P, Flint A, Martens M, Raben A (2003) Effect of sensory perception of foods on appetite and food intake: a review of studies on humans. Int J Obes Relat Metab Disord 27: 1152–1166. 14513063 10.1038/sj.ijo.0802391

[6] Guinard JX, Brun P (1998) Sensory-specific satiety: comparison of taste and texture effects. Appetite 31: 141–157. 9792729 10.1006/appe.1998.0159

[7] Raynor HA, Niemeier HM, Wing RR (2006) Effect of limiting snack food variety on long-term sensory-specific satiety and monotony during obesity treatment. Eat Behav 7: 1–14. 16360618 10.1016/j.eatbeh.2005.05.005

[8] Rolls BJ, Rolls ET, Rowe EA, Sweeney K (1981) Sensory specific satiety in man. Physiol Behav 27: 137–142. 7267792 10.1016/0031-9384(81)90310-3

[9] Rolls ET, Rolls JH (1997) Olfactory sensory-specific satiety in humans. Physiol Behav 61: 461–473. 9089767 10.1016/s0031-9384(96)00464-7

[10] Triscoli C, Croy I, Olausson H, Sailer U (2014) Liking and wanting pleasant odors: different effects of repetitive exposure in men and women. Front Psychol 5: 526. 24910630 10.3389/fpsyg.2014.00526 PMC4038972

[11] Cain WS, Johnson F Jr (1978) Lability of odor pleasantness: influence of mere exposure. Perception 7: 459–465. 704276 10.1068/p070459

[12] Croy I, Maboshe W, Hummel T (2013) Habituation effects of pleasant and unpleasant odors. Int J Psychophysiol 88: 104–108. 23473955 10.1016/j.ijpsycho.2013.02.005

[13] Dawson SJ, Suschinsky KD, Lalumiere ML (2013) Habituation of sexual responses in men and women: a test of the preparation hypothesis of women's genital responses. J Sex Med 10: 990–1000. 23320579 10.1111/jsm.12032

[14] Mcsweeney FK, Roll JM (1998) Do animals satiate or habituate to repeatedly presented reinforcers? Psychonom B Rev 5: 428–442.

[15] Löken LS, Wessberg J, Morrison I, McGlone F, Olausson H (2009) Coding of pleasant touch by unmyelinated afferents in humans. Nat Neurosci 12: 547–548. 19363489 10.1038/nn.2312

[16] Nordin M (1990) Low-threshold mechanoreceptive and nociceptive units with unmyelinated (C) fibres in the human supraorbital nerve. J Physiol 426: 229–240. 2231398 10.1113/jphysiol.1990.sp018135 PMC1189885

[17] Olausson H, Lamarre Y, Backlund H, Morin C, Wallin BG, et al (2002) Unmyelinated tactile afferents signal touch and project to insular cortex. Nat Neurosci 5: 900–904. 12145636 10.1038/nn896

[18] Olausson H, Wessberg J, Morrison I, McGlone F, Vallbo A (2010) The neurophysiology of unmyelinated tactile afferents. Neurosci Biobehav Rev 34: 185–191. 18952123 10.1016/j.neubiorev.2008.09.011

[19] Vallbo A, Olausson H, Wessberg J, Norrsell U (1993) A system of unmyelinated afferents for innocuous mechanoreception in the human skin. Brain Res 628: 301–304. 8313159 10.1016/0006-8993(93)90968-s

[20] Vallbo AB, Olausson H, Wessberg J (1999) Unmyelinated afferents constitute a second system coding tactile stimuli of the human hairy skin. J Neurophysiol 81: 2753–2763. 10368395 10.1152/jn.1999.81.6.2753

[21] Vallbo A, Johansson RS (1984) Properties of cutaneous mechanoreceptors in the human hand related to touch sensation. Hum Neurobiol 3: 3–14. 6330008

[22] Ackerley R, Backlund Wasling H, Liljencrantz J, Olausson H, Johnson RD, et al (2014) Human C-tactile afferents are tuned to the temperature of a skin-stroking caress. J Neurosci 34: 2879–2883. 24553929 10.1523/JNEUROSCI.2847-13.2014 PMC3931502

[23] Morrison I, Bjornsdotter M, Olausson H (2011) Vicarious responses to social touch in posterior insular cortex are tuned to pleasant caressing speeds. J Neurosci 31: 9554–9562. 21715620 10.1523/JNEUROSCI.0397-11.2011 PMC6623148

[24] Bessou P, Burgess PR, Perl ER, Taylor CB (1971) Dynamic properties of mechanoreceptors with unmyelinated (C) fibers. J Neurophysiol 34: 116–131. 5540574 10.1152/jn.1971.34.1.116

[25] Iggo A (1960) Cutaneous mechanoreceptors with afferent C fibres. J Physiol 152: 337–353. 13852622 10.1113/jphysiol.1960.sp006491 PMC1363319

[26] Iggo A, Kornhuber HH (1977) A quantitative study of C-mechanoreceptors in hairy skin of the cat. J Physiol 271: 549–565. 926006 10.1113/jphysiol.1977.sp012014 PMC1353586

[27] Barker DJ, Shepard PD, McDermott KL (1982) Fatigue in cat facial mechanoreceptors. Neurosci Lett 30: 117–122. 6287362 10.1016/0304-3940(82)90282-8

[28] Berridge KC (2009) ‘Liking’ and ‘wanting’ food rewards: brain substrates and roles in eating disorders. Physiol Behav 97: 537–550. 19336238 10.1016/j.physbeh.2009.02.044 PMC2717031

[29] Kringelbach ML, Berridge KC (2009) Towards a functional neuroanatomy of pleasure and happiness. Trends Cogn Sci 13: 479–487. 19782634 10.1016/j.tics.2009.08.006 PMC2767390

[30] Berridge KC, Kringelbach ML (2013) Neuroscience of affect: brain mechanisms of pleasure and displeasure. Curr Opin Neurobiol 23: 294–303. 23375169 10.1016/j.conb.2013.01.017 PMC3644539

[31] Robinson TE, Berridge KC (2001) Incentive-sensitization and addiction. Addiction 96: 103–114. 11177523 10.1046/j.1360-0443.2001.9611038.x

[32] Litman J (2005) Curiosity and the pleasures of learning: Wanting and liking new information. Cognition Emotion 19: 793–814.

[33] Berridge KC (2009) ‘Liking’ and ‘wanting’ food rewards: Brain substrates and roles in eating disorders. Physiol Behav 97: 537–550. 19336238 10.1016/j.physbeh.2009.02.044 PMC2717031

[34] Berridge KC (2003) Pleasures of the brain. Brain Cogn 52: 106–128. 12812810 10.1016/s0278-2626(03)00014-9

[35] Mela DJ (2006) Eating for pleasure or just wanting to eat? Reconsidering sensory hedonic responses as a driver of obesity. Appetite 47: 10–17. 16647788 10.1016/j.appet.2006.02.006

[36] Berridge KC (1996) Food reward: brain substrates of wanting and liking. Neurosci Biobehav Rev 20: 1–25. 8622814 10.1016/0149-7634(95)00033-b

[37] Berridge KC, Robinson TE (2003) Parsing reward. Trends Neurosci 26: 507–513. 12948663 10.1016/S0166-2236(03)00233-9

[38] Triscoli C, Olausson H, Sailer U, Ignell H, Croy I (2013) CT-optimized skin stroking delivered by hand or robot is comparable. Front Behav Neurosci 7: 208. 24391564 10.3389/fnbeh.2013.00208 PMC3866892

[39] Berridge KC, Robinson TE, Aldridge JW (2009) Dissecting components of reward: ‘liking’, ‘wanting’, and learning. Curr Opin Pharmacol 9: 65–73. 19162544 10.1016/j.coph.2008.12.014 PMC2756052

[40] Rolls ET, McCabe C (2007) Enhanced affective brain representations of chocolate in cravers vs. non-cravers. Eur J Neurosci 26: 1067–1076. 17714197 10.1111/j.1460-9568.2007.05724.x

[41] Finlayson G, King N, Blundell JE (2007) Is it possible to dissociate ‘liking’ and ‘wanting’ for foods in humans? A novel experimental procedure. Physiol Behav Volume 90: 36–42. 10.1016/j.physbeh.2006.08.020 17052736

[42] Havermans RC (2011) “You Say it's Liking, I Say it's Wanting …”. On the difficulty of disentangling food reward in man. Appetite 57: 286–294. 21635928 10.1016/j.appet.2011.05.310

[43] Carver CS, White TL (1994) Behavioral inhibition, behavioral activation, and affective responses to impending reward and punishment: The BIS/BAS Scales. J Pers Soc Psychol 67: 319–333.

[44] Gard DE (2006) Anticipatory and consummatory components of the experience of pleasure: a scale development study. J Res Pers 40: 1086–1102.

[45] Peck J, Childers TL (2003) Individual Differences in Haptic Information Processing: The “Need for Touch” Scale. J Consum Res 30: 430–442.

[46] Gray JA, McNaughton N (1982) The Neuropsychology of Anxiety: An Enquiry into the Functions of the Septo-Hippocampal System. Oxford: Oxford University Press.

[47] Hickey C, Chelazzi L, Theeuwes J (2010) Reward Guides Vision when It's Your Thing: Trait Reward-Seeking in Reward-Mediated Visual Priming. PLOS ONE 5: e14087. 21124893 10.1371/journal.pone.0014087 PMC2990710

[48] Cornell C, Rodin J, Weingarten H (1989) Stimulus-induced eating when satiated. Physiol Behav 45: 695–704. 2780836 10.1016/0031-9384(89)90281-3

[49] Rolls BJ, Castellanos VH, Halford JC, Kilara A, Panyam D, et al (1998) Volume of food consumed affects satiety in men. Am J Clin Nutr 67: 1170–1177. 9625090 10.1093/ajcn/67.6.1170

[50] Ackerley R, Carlsson I, Wester H, Olausson H, Backlund et al (2014) Touch perceptions across skin sites: differences between sensitivity, direction discrimination and pleasantness. Front Behav Neurosci 8: 54. 24600368 10.3389/fnbeh.2014.00054 PMC3928539

[51] Löken LS, Evert M, Wessberg J (2011) Pleasantness of touch in human glabrous and hairy skin: order effects on affective ratings. Brain Res 1417: 9–15. 21907328 10.1016/j.brainres.2011.08.011

[52] Hetherington MM, Foster R, Newman T, Anderson AS, Norton G (2006) Understanding variety: tasting different foods delays satiation. Physiol Behav 87: 263–271. 16405929 10.1016/j.physbeh.2005.10.012

[53] Rolls BJ, Fedoroff IC, Guthrie JF, Laster LJ (1990) Foods with different satiating effects in humans. Appetite 15: 115–126. 2268137 10.1016/0195-6663(90)90044-9

[54] Grice RG, Hunter JJ (1964) Stimulus intensity effects depend upon the type of experimental design. Psychol Rev 71: 247–256. 14183610 10.1037/h0047547

[55] Blakemore SJ, Frith CD, Wolpert DM (1999) Spatio-temporal prediction modulates the perception of self-produced stimuli. J Cogn Neurosci 11: 551–559. 10511643 10.1162/089892999563607

